# *Streptococcus anginosus*: a stealthy villain in deep odontogenic abscesses

**DOI:** 10.1007/s10266-022-00763-z

**Published:** 2022-11-08

**Authors:** Jussi Furuholm, Johanna Uittamo, Niina Rautaporras, Hanna Välimaa, Johanna Snäll

**Affiliations:** 1 Department of Oral and Maxillofacial Diseases, University of Helsinki, P.O. Box 41, 00014 Helsinki, Finland; 2 Department of Oral and Maxillofacial Diseases, Helsinki University Hospital, P.O. Box 447, 00029 Helsinki, Finland; 3 Meilahti Vaccine Research Center MeVac, Department of Infectious Diseases, University of Helsinki and Helsinki University Hospital, Helsinki University Hospital, P.O. Box 700, FI-00029 Helsinki, Finland

**Keywords:** Odontogenic infection, Hospital care, Intensive care, Oral microbiology, Streptococcal infections

## Abstract

Odontogenic infections (OIs) occasionally spread to deep facial and neck tissues. Our study aimed to explore the role of *Streptococcus anginous* group (SAG) in these severe OIs. A retrospective study of patients aged ≥ 18 years who required hospital care for acute OI was conducted. We analysed data of OI microbial samples and recorded findings of SAG and other pathogens. These findings were compared with data regarding patients’ prehospital status and variables of infection severity. In total, 290 patients were included in the analyses. The most common (49%) bacterial finding was SAG. Other common findings were *Streptococcus viridans* and *Prevotella* species, *Parvimonas micra,* and *Fusobacterium nucleatum*. Infection severity variables were strongly associated with SAG occurrence. Treatment in an intensive care unit was significantly more common in patients with SAG than in patients without SAG (*p* < 0.001). In addition, SAG patients expressed higher levels of C-reactive protein (*p* = 0.001) and white blood cell counts (*p* < 0.001), and their hospital stays were longer than those of non-SAG patients (*p* = 0.001). SAG is a typical finding in severe OIs. Clinical features of SAG-related OIs are more challenging than in other OIs. Early detection of SAG, followed by comprehensive infection care with prompt and careful surgical treatment, is necessary due to the aggressive behaviour of this dangerous pathogen.

## Introduction

Odontogenic infections (OIs) can spread deeper into the surrounding tissues, requiring hospitalization, and potentially posing a life-threatening risk [1–3]. Behind more severe deep neck infections lie certain predictors, such as psychiatric disorder, alcohol abuse, and diabetes mellitus [4–6], although previously healthy patients may require hospital care due to an OI, as well [2, 7]. Mandibular molars are the most common sources of severe OIs [8, 9], and these arise from apical periodontitis in particular [10]. In addition, deep infection may occur after tooth removal to treat acute or subacute local infection symptoms [11]. Early management of the dental infection focus by tooth removal or root canal treatment effectively reduces dispersion of the infection, however, occasionally, the pathogenic microbes invade deeper spaces, causing infection complications such as pneumonia, septicaemia, and endocarditis [12–14].

The complexity of the oral microbiome is well known, with over 500–700 bacterial species identified [15–17]. Both cariogenic and periodontal pathogens have been detected in heart valve specimens, suggesting a causative relationship between dental infections and cardiovascular diseases [18, 19]. OIs and dental infectious diseases in general are polymicrobial and often caused by anaerobic and facultative bacteria [20, 21]. *Streptococcus viridans* species, including *Streptococcus anginosus* group (SAG)—formerly and popularly known as *Streptococcus milleri* group, have been identified as the most frequent bacteria in head and neck infections of odontogenic origin, resulting in increasing concern for antibiotic resistance, particularly for penicillin and clindamycin [22–24]. This emphasizes the importance of timely local treatment of the dental focus [5, 25].

Members of the SAG—*Streptococcus intermedius*, *Streptococcus constellatus*, and *Streptococcus anginosus*—share common traits regarding clinical associations but differ slightly in their abscess formation capacity [26]. Recent and earlier findings suggest *Streptococcus anginosus* group as a key factor in odontogenic descending necrotizing mediastinitis, pulmonary infections, and brain abscesses [27–29], while the presence of SAG has also been reported in many other morbidities such as skin and soft tissue infections, genitourinary infections and liver abscesses [30, 31]. However, the SAG organisms are categorized as commensals in the oropharynx as well as in the gastrointestinal and genitourinary tracts [32].

The aim of our study was to clarify the occurrence of SAG in deep OIs and to evaluate the clinical features and severity of these infections in relation to OIs caused by other microbes. Our hypothesis was that infections caused by SAG are associated with more complicated clinical features.

## Patients and methods

### Study design

Electronic health records of all acute maxillofacial infection patients treated at the Töölö Hospital Emergency Department between the years 2015 and 2019 were retrospectively reviewed, and data for study variables were retrieved from electronic patient records of each patient.

### Inclusion and exclusion criteria

Patients aged ≥ 18 years who required treatment and hospital stay for acute and deep OI (*i.e.*, abscess or cellulitis of facial or neck region of dental origin) were included in the present study. All infections were confirmed as odontogenic by oral and maxillofacial surgeons. Patients with infection of unknown origin or other than odontogenic focus as a reason for maxillofacial infection were excluded from the analyses. Patients without any microbial finding in bacterial culture were also excluded.

### Study variables

Occurrence of SAG and other microbiological findings were recorded based on the routine bacterial culture reports released from Helsinki University Hospital Laboratory Services (HUSLAB). Patient’s prehospital and infection severity variables were compared between patients with and without SAG findings.

Age, sex, current smoking, excess alcohol consumption or regular use of drugs, and history of immunosuppressive disease or medications, or both, and duration of symptoms prior to hospital care were considered in the analyses as prehospital variables. Excess alcohol consumption was considered to be ≥ 12 doses per week for women and ≥ 23 doses for men; one alcohol dose was 12 g of pure alcohol.

To evaluate infection severity, need for treatment in an intensive care unit (ICU), level of C-reactive protein (CRP), white blood cell (WBC) count and tympanic body temperature at hospital admission, length of hospital stay (LOHS) in an ICU and in hospital, and occurrence of a distant infection (i.e., different distant infections and infection complications) were analysed. Multivariate analyses were conducted for need for ICU treatment, LOHS, and CRP at hospital admission.

Additionally, types of specific infection complications and distant infections were reported.

### Statistical analysis

IBM SPSS for Macintosh (version 27.0, IBM Corp., Armonk, NY, USA) software package was used for statistical analyses. Categorical variables were cross-tabulated and analysed with Pearson’s Chi-squared test or Fisher’s exact test if expected values were below 5. Student’s t-test and Mann–Whitney *U* test were used to compare differences between study groups in continuous variables. Pairwise comparisons were performed as post hoc analyses for Pearson’s Chi-squared test using *Z* test and Dunn’s (1964) procedure for Kruskal–Wallis *H* test, both with a Bonferroni correction for multiple comparisons. Treatment in ICU, LOHS, and CRP level were separately selected as outcome variables for binomial logistic regression analysis; age, sex, current smoking, excess alcohol consumption or regular use of drugs, history of immunosuppressive disease or medication, or both, origin of infection in mandible, and SAG-positive microbial sample were selected as independent variables. LOHS and CRP level were dichotomized by group median, and age was categorized into tertiles. *P* values ˂ 0.05 were considered significant throughout the study.

## Results

Data from altogether 357 patients with OI were collected from the electronic patient records. Of these patients, a bacterial sample had been obtained and microbial growth reported for 290 patients, who subsequently formed the final study population.

**Fig. 1:**
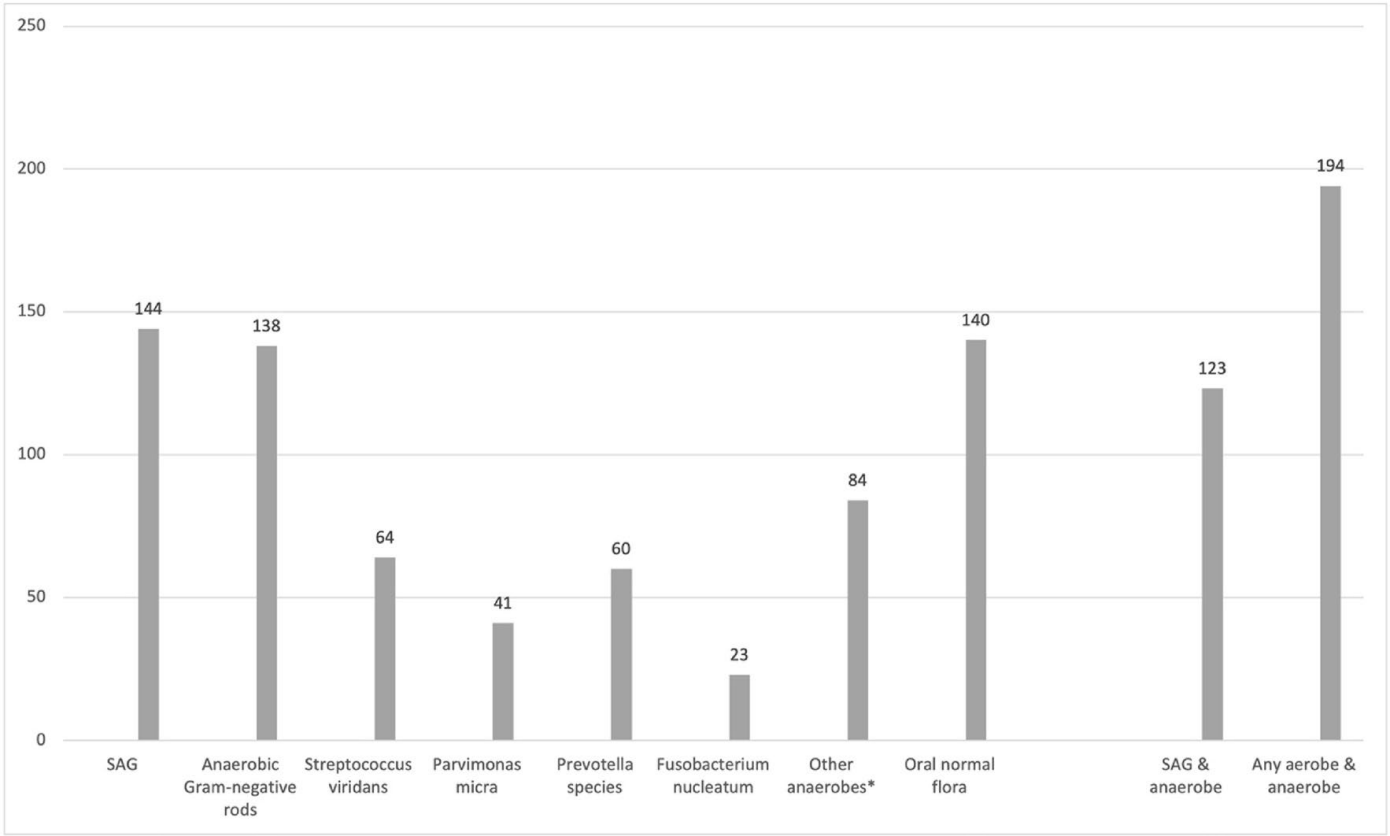
Frequencies of different bacterial species in 290 patients. *Excluding Gram-negative rods, *Parvimonas micra, Prevotella* species, *Fusobacterium nucleatum*. *SAG Streptococcus anginosus* group

In 194 subjects (67%), the bacterial culture finding was a mixed finding of aerobic and anaerobic bacteria. Aerobic bacteria alone were reported in 60 cultures (21%) and anaerobic bacteria alone in 55 cultures (19%). The most common bacterial finding was SAG, which was found in 49% of patients. In 123 cases (42%), SAG was diagnosed with anaerobic bacteria. Other common findings were normal oral microbial flora (48%) and anaerobic Gram-negative rods (47%, Fig. 1). Among other common findings were *Streptococcus viridans* group and *Prevotella* species, *Parvimonas micra,* and *Fusobacterium nucleatum*.

In only few samples, findings not representative of normal oral flora were discovered. Among these were the aerobic cocci betahaemolytic streptococci (*n* = 4; 1%) and *Staphylococcus aureus* (*n* = 4; 1%), aerobic Gram-negative rods *Klebsiella pneumoniae* (*n* = 3; 1%) and *Enterobacter cloacae* (*n* = 3; 1%), and anaerobic species of the *Bacteroides fragilis* group (*n* = 3; 1%).

Of prehospital variables, mandibular odontogenic infection focus was more strongly associated with SAG than focus in the maxilla (*p* = 0.012, Table 1). Additionally, there was a significant association between SAG and tooth removal before hospitalization (*p* = 0.041). Antibiotic treatment was administered to all but one patient. In most patients (*n* = 269, 93%), metronidazole was combined with cephalosporin or penicillin. Clindamycin was administered to 8 patients (3%). In 35 patients (12%), antibiotic treatment was altered during hospital stay according to clinical and microbiological findings.

**Table 1:** Associations between explanatory variables and patients with and without a positive *Streptococcus anginosus* group sample

	No. of patients with *Streptococcus anginosus* (*n* = 144)		No. of patients without *Streptococcus anginosus* (*n* = 146)			
	*n*	% of *n*	*n*	% of *n*	*p*-value	Effect size if significant
Age (years)						
Mean ± SD	46.4 ± 18.95		47.6 ± 16.75		0.598	
Median (min − max)	46 (18–96)					
≤ 46 (*n* = 147)	77	52	70	48	0.347	
> 46 (*n* = 143)	67	47	76	53		
Sex						
Men (*n* = 174)	82	47	93	53	0.291	
Women (*n* = 116)	62	53	54	47		
Smoking						
Yes (*n* = 83)	37	45	46	55	0.274	
No (*n* = 207)	107	52	100	48		
Heavy alcohol use						
Yes (*n* = 28)	16	57	12	43	0.404	
No (*n* = 262)	128	49	134	51		
Immunocompromised condition due to disease and/or medication						
Yes (*n* = 51)	28	55	23	45	0.409	
Diabetes (n = *24)*	*14*	*58*	*10*	*42*	*0.375*	
No (*n* = 239)	116	48	126	52		
Site of infection						
Mandible (*n* = 256)	134	52	122	48	**0.012**	0.148
Maxilla (*n* = 34)	10	29	24	71		
Tooth removal before hospitalization						
Yes (*n* = 104)	60	58	44	42	**0.041**	0.120
No (*n* = 186)	84	45	102	55		

*p* < 0.05 values are in bold

Infection severity variables were strongly associated with SAG occurrence. ICU treatment was significantly more common in patients with SAG than in patients without SAG (*p* < 0.001). Compared with non-SAG patients, SAG patients expressed higher levels of CRP (*p* < 0.001) and WBC counts (*p* = 0.001), and their LOHS was longer (*p* = 0.001), as presented in Table 2.

**Table 2:** Associations between infection severity variables and patients with and without a positive *Streptococcus anginosus* group sample

	No. of patients with *Streptococcus anginosus* (*n* = 144)		No. of patients without *Streptococcus anginosus* (*n* = 146)			
	*n*	% of *n*	*n*	% of *n*	*p*-value	Effect size if significant
ICU treatment						
Yes (*n* = 83)	58	70	25	30	** < 0.001**	0.256
No (*n* = 207)	86	41	121	59		
CRP level at hospital admission (mg/L)						
Mean ± SD	171.5 ± 99.53		136.4 ± 80.52		** < 0.001**	0.393
Median (min − max)	134.5 (6–565)					
≤ 134.5 (*n* = 145)	62	43	83	57	**0.019**	0.138
> 134.5 (*n* = 145)	82	57	63	43		
WBC count at hospital admission (10^9^/L)						
Mean ± SD	14.4 ± 5.47		12.5 ± 4.30		**0.001**	0.380
Median (min − max)	12.6 (1.3–35.9)					
≤ 12.6 (*n* = 144)	63	44	81	56	**0.046**	0.117
> 12.6 (*n* = 146)	81	56	65	44		
Tympanic temperature						
< 38.0 °C (*n* = 216)	103	48	113	52	0.252	
≥ 38.0 °C (*n* = 74)	41	55	33	45		
Length of hospital stay (days)						
Frequency*	*n* = 140		*n* = 144			
Mean ± SD	4.4 ± 4.62		2.9 ± 2.68		**0.001**	0.391
Median (min − max)	2 (< 1–37)					
≤ 2 (*n* = 147)	58	39	89	61	** < 0.001**	0.204
> 2 (*n* = 137)	82	60	54	40		
	*3 deceased and data missing for 3					
Infection complication or distant infection						
Yes (*n* = 22)	14	64	8	36		
No (*n* = 268)	130	48	138	52	0.172	

*p* < 0.05 values are in bold

Distant infections and/or other infection complications occurred in 22 of 290 patients (8%, Table 2). Complications were more common in patients with SAG than in other patients (*n* = 14, 64% vs. *n* = 8, 36% of infection complications), however, the difference was not statistically significant (*p* = 0.153). Bacterial blood culture sample was collected and analysed in 125 patients. Of the 12 patients with septicaemia, 10 were SAG-positive and two were SAG-negative (*p* = 0.042). Other distant infections and/or other infection complications in 13 patients were pneumonia (*n* = 10), endocarditis (*n* = 2), necrotizing fasciitis (*n* = 2), urosepsis (*n* = 1), and embolic renal infarction (*n* = 1); of these patients, 8 (62%) were SAG-positive and 5 (38%) SAG-negative (*p* = 0.354). Three of the patients died; all were SAG-positive.

According to binomial logistic regression analyses (Table 3), patients with a SAG-positive microbial sample were 3.4 times more likely to need treatment in an ICU (*p* < 0.001). Smoking (odds ratio, OR = 2.1, *p* = 0.024) and origin of OI in mandible (OR = 5.8, *p* = 0.021) added independently to the odds for need for ICU treatment. Odds for longer hospital stay were 2.3-fold (*p* < 0.001), for higher CRP values 1.9-fold (*p* < 0.010), and for higher than median WBC counts 1.7-fold (*p* = 0.0351) for SAG-positive patients relative to SAG-negative patients.

**Table 3:** Results of binomial logistic regression analyses

		OR	95% CI for OR		
			Lower	Upper	*p*-value
A	Need for ICU treatment				
	Age, lowest tertile (ref.)				0.828
	Age, middle tertile	0.822	0.420	1.609	0.568
	Age, highest tertile	0.848	0.426	1.687	0.638
	Sex, male	1.535	0.843	2.797	0.161
	Smoking	2.071	1.099	3.904	**0.024**
	Heavy alcohol use	1.102	0.420	2.892	0.844
	Immunocompromised condition due to disease and/or medication	0.601	0.269	1.344	0.215
	Origin of infection in mandible	5.759	1.302	25.481	**0.021**
	SAG-positive	3.393	1.907	6.037	** < 0.001**
B	Higher than median LOHS*				
	Age, lowest tertile (ref.)				0.859
	Age, middle tertile	0.976	0.542	1.757	0.935
	Age, highest tertile	1.145	0.626	2.096	0.660
	Sex, male	1.251	0.750	2.088	0.391
	Smoking	1.052	0.602	1.841	0.858
	Heavy alcohol use	0.936	0.386	2.268	0.883
	Immunocompromised condition due to disease and/or medication	1.405	0.720	2.742	0.319
	Origin of infection in mandible	1.163	0.541	2.498	0.699
	SAG-positive	2.302	1.411	3.756	** < 0.001**
	*n = 284, 3 deceased and data missing for 3				
C	Higher than median CRP				
	Age, lowest tertile (ref.)				0.897
	Age, middle tertile	1.116	0.624	1.994	0.712
	Age, highest tertile	0.980	0.544	1.766	0.947
	Sex, male	1.487	0.900	2.459	0.122
	Smoking	0.924	0.533	1.601	0.778
	Heavy alcohol use	0.666	0.279	1.591	0.361
	Immunocompromised condition due to disease and/or medication	0.797	0.415	1.529	0.494
	Origin of infection in mandible	0.773	0.363	1.643	0.503
	SAG-positive	1.886	1.165	3.055	**0.010**
D	Higher than median WBC				
	Age, lowest tertile (ref.)				0.832
	Age, middle tertile	1.006	0.562	1.798	0.985
	Age, highest tertile	0.855	0.474	1.542	0.604
	Sex, male	1.363	0.827	2.246	0.224
	Smoking	1.325	0.763	2.301	0.318
	Heavy alcohol use	1.391	0.575	3.365	0.464
	Immunocompromised condition due to disease and/or medication	0.663	0.343	1.280	0.220
	Origin of infection in mandible	1.068	0.502	2.271	0.865
	SAG-positive	1.677	1.037	2.714	**0.035**

*p* < 0.05 values are in bold

*LOHS* length of hospital stay, *OR* odds ratio, *CI* confidence interval

## Discussion

We examined the occurrence of SAG in deep OIs and the clinical features and severity of these infections in relation to OIs caused by other microbes. Our hypothesis that infections caused by SAG are associated with more complicated clinical features was confirmed.

SAG was present in nearly half of the patients, and it was the most common bacterial finding. SAG-associated infections were significantly more complicated than those without SAG. Need for ICU treatment was significantly more likely for SAG-positive patients (OR = 3.4, *p* < 0.001). Higher levels of CRP, WBC, and LOHS were associated with SAG. These findings imply that SAG infections are complicated, severe, and life-threatening. The typical nature of SAG-associated OIs differs from OIs caused by other pathogens.

**Fig. 2:**
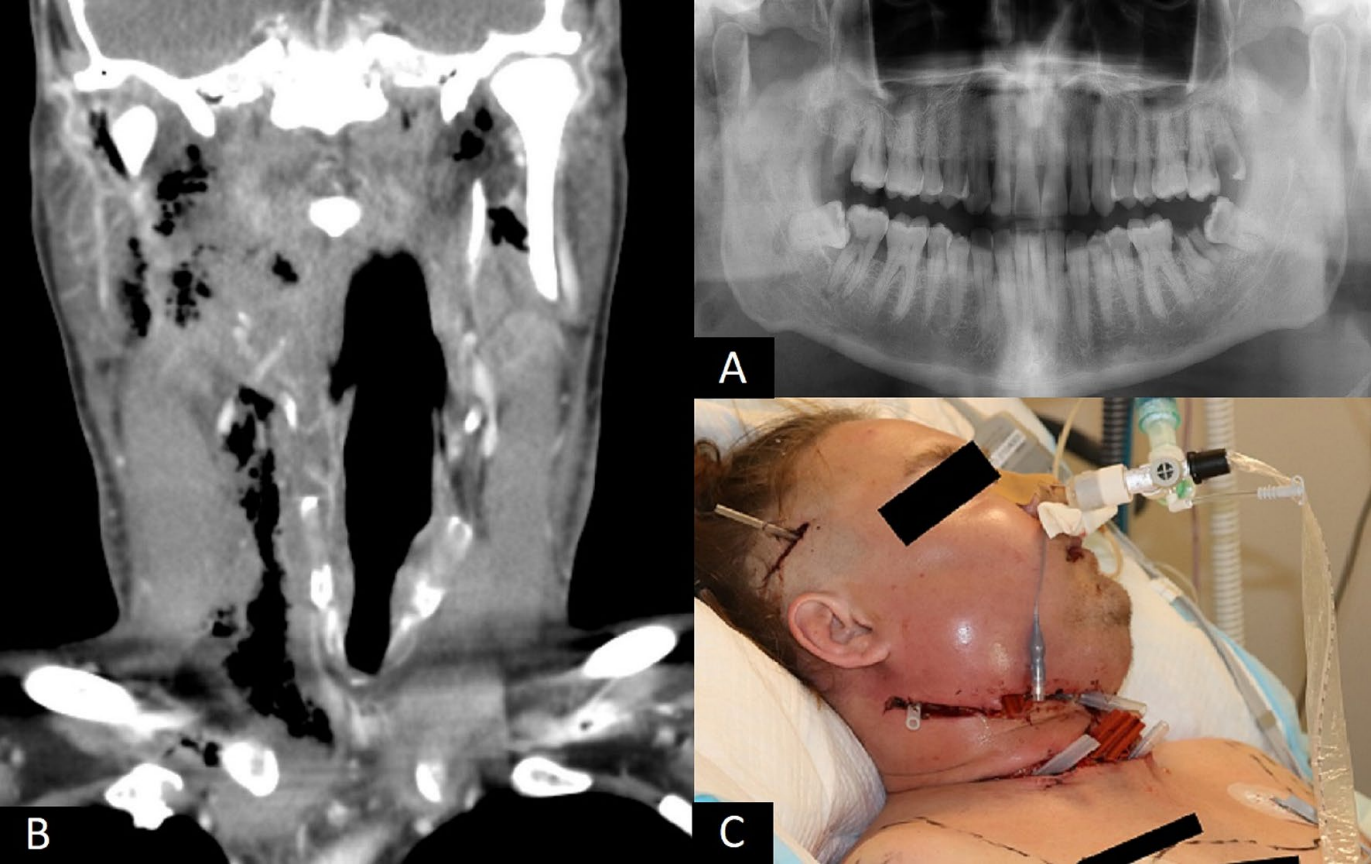
A 40-year-old male smoker without underlying diseases had had toothache in the right lower jaw for 3 days before onset of significant swelling and fever (39.2 °C). At hospital admission, C-reactive protein level was 253 mg/L and white blood cell count was 18.2 10^9^/L. The patient underwent three wide surgical interventions to subside the deep oral, facial, and neck infection. Bacterial culture from the pus sample showed *Streptococcus anginosus* and mixed anaerobic flora. Blood culture was also positive (anaerobic Gram-positive coccus and anaerobic Gram-negative rod). **A.** Dental panoramic tomography radiograph showed the right mandibular third molar with periapical lesion as the main infection focus. **B.** Computer tomography imaging at hospital admission demonstrated bilateral gas formation in neck tissues from the level of skull base to the upper mediastinal space at the right side. The airway was also deviated. **C.** Extensive neck approaches were required, including temporal and upper mediastinal drainages. Swelling and tightness in buccal and periorbital areas remained significant after the third surgery. The overall length of hospital stay was 32 days. The patient eventually recovered well from the infection. A written consent was obtained from the patient for use of the image

In principle, SAG organisms are classified as commensals. However, it is essential to emphasize the clinical importance of the aggressive behaviour of SAG in severe OIs. SAG bacteria are able to cause infections if they gain access through mucosa to sterile sites, i.e., the underlying tissue or blood, and can, therefore, be viewed as opportunistic pathogens. It has previously been shown that SAG-related OIs are linked to the most severe disease, with difficulties in swallowing and opening the mouth [33]. In spreading OIs, SAG behaviour is known to be typically complicated and abscess-forming [34]. Our findings confirm the clinical significance of this aggressive pathogen (Fig. 2). However, despite the clinically notable typical infection features, the true pathogenic potential of SAG remains to be elucidated [35]. A recent study by Ismail et al*.* [36] clarified SAG occurrence in different infections in children and adolescents and discovered that *S. intermedius* was more commonly present in head and neck infections than other SAG species. Thus, differences exist in infection locations and SAG species.

A suggested novel trait in the pathogenicity of *S. anginosus* may be its ability to produce bacteriocin designated as Angicin, which enhances membrane permeabilization [37], inhibits the growth of closely related species [38], and enables compartmental abscess formation. Acid tolerance exhibited by the micro-organism is another factor that may facilitate chronic inflammation and abscess development [39]. Ardent fever, chills, and systemic toxaemia have been observed in severe SAG infections; upper airway obstructions and necrotizing pneumonia are possible outcomes of abscess formation [33, 40]. Our results show that in SAG-related OI patients the airways are more often compromised, the infection parameters reflect a more severe infection, and the treatment of these infections takes longer than in other OIs. The potentially fatal characteristics of SAG infections emphasize the importance of early detection of these OIs.

To identify SAG infections without unnecessary delay their clinical features should be recognized. These include rapid and aggressive progression. According to our study, high CRP and WBC level as well as fever at hospital admission are typical for SAG infections. Gas bubble formation in radiological imaging is indicative of SAG [27]. The surgeon should consider SAG at an early stage and treat these infections with precision. Sufficient surgical treatment with wide approaches and careful abscess drainage is necessary to halt the progression of SAG infection. SAG is typically identified in abscesses as part of polymicrobial infections with anaerobes. In our study, SAG was identified concomitantly with anaerobic bacteria in 85% of the samples with SAG. This underscores the need for abscess drainage as a crucial component of successful treatment. Furthermore, the simultaneous removal of odontogenic infection focus is known to shorten LOHS compared with tooth extraction after initial infection treatment [25]. Compartmental, tissue-disrespectful progression is typical of SAG, thus, close monitoring of the patient and, if necessary, repeated evaluation of the abscess chambers is essential.

Compared with figures from previous reports [13, 41], infection complications and distant infections were relatively scarce, occurring in 8.3% of patients. The most common of these were septicaemia and pneumonia, whereas necrotizing fasciitis and endocarditis were each observed in only two patients. The rare occurrence of the most severe infections highlights the typical pattern of OIs; even deep infections requiring intensive care are limited to the neck area if treatment is effective and started in time. On the other hand, SAG is often associated with infection complications and distant infections, especially with septicaemia. All three deceased patients here were SAG-positive. These findings underscore the life-threatening features of SAG in OI patients.

Our research shows a clear association between the site of infection and the presence of SAG; OIs originating from the mandible occurred significantly more often in conjunction with SAG than maxillary infections. In general, most severe OIs are known to originate more often from the lower molars [7, 9]. We also observed that tooth removal prior to hospitalization was significantly more common in patients with SAG than those without (56% vs. 44%, *p* = 0.041). The finding suggests that previous tissue damage may predispose to the development of severe SAG-associated OIs.

The importance of immune defense and previous diseases is also worth considering. Currently, however, there are no reports showing that microbial findings would be altered in severe OIs compared to milder infections according to patients’ systemic condition, although the proportion of patients with underlying systemic disease has been reported to have increased [2, 41]. Our earlier studies have shown that severe OIs occur most often in previously healthy adults [11, 42]. Dental procedures including extraction and root canal treatment disrupt the mucosal barrier allowing introduction of mucosal opportunistic bacteria to normally sterile tissue, are local oral risk factors for severe OIs [10, 11]. Furthermore, ineffective early treatment of OI may increase the risk of a severe OI [42]. The findings of the present study are in line with these previous results as immunosuppression was not associated with SAG finding (Table 1). Additionally, only 18% of patients had underlying immunosuppressive medication or disease. In conclusion, the risk of these infections seems to be primarily associated with local factors such as dental treatment procedures, delayed or inadequate dental treatment, or increased systemic susceptibility to infection complications, rather than altered microbial flora in certain systemic conditions.

In addition to drainage and treatment of odontogenic focus, antimicrobial treatment is often required for successful treatment of purulent OIs. These infections are usually polymicrobial, consisting primarily of SAG or other Viridans streptococci and anaerobic bacteria. Other microbes are rare findings, as observed also in our study. Yet another important aspect to consider in choosing the antimicrobial is the local resistance situation. Fortunately, in Finland, SAG and other Viridans streptococci have remained fairly sensitive to penicillin, with only a 1–5% resistance rate, and oral anaerobes remain highly susceptible to metronidazole [43]. In contrast, resistance to clindamycin of oral streptococci has recently slightly increased and needs to be monitored. Therefore, the Finnish Current Care Guideline recommends primarily using penicillin for oral streptococci and metronidazole for anaerobic bacteria, which may be beta-lactamase producers [44]. Broader spectrum cephalosporins or clindamycin are recommended instead of penicillin in case of penicillin allergy. If metronidazole is contraindicated, clindamycin can be combined with penicillin to cover anaerobes, if needed. If the above combinations are contraindicated, broad-spectrum amoxicillin-clavulanic acid may be used as monotherapy.

For the retrospective study design, some of the patients was excluded from the study because of unavailable bacterial cultures. In addition, PCR testing to determine the species of bacteria is not performed in our unit routinely, thus, the determination of bacteria was based on culture only. Data of patients treated in primary or secondary health care was unavailable; our data were collected from hospitalized patients. A prospective study design would refine these parameters. New and effective treatments for SAG-related infections should be explored in future studies.

## Conclusions

SAG is a typical finding in severe OIs. Clinical features of SAG-related OIs are more aggressive than in other OIs. Early detection of SAG, followed by comprehensive infection care with prompt and careful surgical treatment, is essential due to the aggressive behaviour of this stealthy pathogen.

## Data Availability

The datasets generated in the study are not publicly available as this requires research ethics approval and participant consent. A summary of final findings will be available from the corresponding author on reasonable request.
